# VDJPipe: a pipelined tool for pre-processing immune repertoire sequencing data

**DOI:** 10.1186/s12859-017-1853-z

**Published:** 2017-10-11

**Authors:** Scott Christley, Mikhail K. Levin, Inimary T. Toby, John M. Fonner, Nancy L. Monson, William H. Rounds, Florian Rubelt, Walter Scarborough, Richard H. Scheuermann, Lindsay G. Cowell

**Affiliations:** 1 Department of Clinical Sciences, UT Southwestern Medical Center, Dallas, TX 75390 USA; 2 Bank of America Corporate Center, Charlotte, NC 28202 USA; 3 Texas Advanced Computing Center, Austin, TX 78758-4497 USA; 4 Department of Neurology and Neurotherapeutics, UT Southwestern Medical Center, Dallas, TX 75390 USA; 5 Department of Immunology, UT Southwestern Medical Center, Dallas, TX 75390 USA; 6 Department of Microbiology and Immunology, Stanford University School of Medicine, Stanford, CA 94305 USA; 7 J. Craig Venter Institute, La Jolla, CA 92037 USA; 8 Department of Pathology, University of California, San Diego, CA 92093 USA; 9 La Jolla Institute for Allergy & Immunology, La Jolla, CA 92037 USA

**Keywords:** Rep-seq, Immune repertoire analysis, Bioinformatics

## Abstract

**Background:**

Pre-processing of high-throughput sequencing data for immune repertoire profiling is essential to insure high quality input for downstream analysis. VDJPipe is a flexible, high-performance tool that can perform multiple pre-processing tasks with just a single pass over the data files.

**Results:**

Processing tasks provided by VDJPipe include base composition statistics calculation, read quality statistics calculation, quality filtering, homopolymer filtering, length and nucleotide filtering, paired-read merging, barcode demultiplexing, 5′ and 3′ PCR primer matching, and duplicate reads collapsing. VDJPipe utilizes a pipeline approach whereby multiple processing steps are performed in a sequential workflow, with the output of each step passed as input to the next step automatically. The workflow is flexible enough to handle the complex barcoding schemes used in many immunosequencing experiments. Because VDJPipe is designed for computational efficiency, we evaluated this by comparing execution times with those of pRESTO, a widely-used pre-processing tool for immune repertoire sequencing data. We found that VDJPipe requires <10% of the run time required by pRESTO.

**Conclusions:**

VDJPipe is a high-performance tool that is optimized for pre-processing large immune repertoire sequencing data sets.

## Background

The ability to mount an effective immune response and subsequently develop specific immunity relies on the presence of a diverse repertoire of antigen receptors. In jawed vertebrates, the genes encoding antibodies and antigen receptors are somatically generated in lymphocytes through a DNA recombination process, V(D)J recombination, which assembles variable (V), diversity (D), and joining (J) gene segments into genes. The diversity of gene sequences generated by V(D)J recombination is huge (estimated at >10^15^) as a result of varying combinations of V, D, and J gene segments, as well as sequence modifications (e.g., exonucleolytic trimming and non-templated nucleotide addition) at the junctions of rearranged gene segments [1, 2]. In recent years, the increased sample coverage of next generation sequencing technologies has significantly enhanced our ability to obtain detailed characterizations of immune repertoires, as there can easily be more than 10^6^ unique sequences for each receptor type [3, 4].

Analysis of immune repertoire sequencing data shares some pre-processing tasks with other next generation sequencing data [5, 6], but it also has its own unique characteristics, including 5′ and 3′ PCR primer targeting, complex multi-level barcode demultiplexing, and duplicate sequence read collapsing. Table 1 provides a list of immune repertoire analysis tools with their pre-processing capabilities. The set of tools can be divided into three main categories: 1) those providing a workflow from raw data to V, D, and J assignment, 2) those specializing in specific pre-processing operations, and 3) those providing generic pre-processing operations. We excluded web analysis portals such as VDJServer and ARGalaxy, as they provide web-based access to other tools. We indicate each tool’s category (1, 2 or 3) in Table 1. All of the tools in the first category provide some limited pre-processing capability, though this tends to be restricted to barcode demultiplexing and length and quality filtering. Category 2 tools specialize in processing unique molecular identifiers (UMI), which is a strategy to overcome PCR amplification bias and sequencing error [7]. The tools in Category 3, which includes VDJPipe, provide the broadest capability.

**Table 1:** List of analysis tools providing pre-processing functions

Tool	Category	Composition statistics calculation	Merging paired-end reads	Barcode demultiplexing	PCR primer removal	Length filtering	Quality filtering	Homopolymer filtering	UMI consensus determination	Collapsing duplicate reads
AbStar [16]	1	No	Yes	Yes	No	Yes	Yes	No	No	No
Decombinator [17]	2	No	No	Yes	No	No	No	No	Yes	No
Ig-HTS cleaner [18]	3	Yes	No	Yes	Yes	Yes	Yes	No	No	No
IgRepertoire constructor [19]	1	No	Yes	Yes	No	No	No	No	Yes	No
ImmunediveRsity [20]	1	No	No	No	No	Yes	Yes	No	No	No
iMonitor [21]	1	No	Yes	No	No	Yes	Yes	No	No	No
IMSEQ [22]	1	No	Yes	Yes	No	Yes	Yes	No	No	No
MIGEC [7]	2	No	Yes	Yes	No	No	Yes	No	Yes	No
pRESTO [13]	3	No	Yes	Yes	Yes	Yes	Yes	No	Yes	Yes
RTCR [23]	1	No	No	Yes	No	Yes	Yes	No	No	No
VDJPipe	3	Yes	Yes	Yes	Yes	Yes	Yes	Yes	No	Yes
TCRklass [24]	1	No	Yes	No	No	Yes	Yes	No	No	No
Abmining [25]	3	No	Yes	Yes	No	Yes	Yes	Yes	No	No
ClonoCalc [26]	1	No	Yes	Yes	No	No	No	No	No	No

VDJPipe is a flexible, high-performance tool for performing a variety of pre-processing operations on immune repertoire sequencing data. VDJPipe efficiently performs all of these pre-processing tasks within a user-defined pipeline that only requires a single pass over large sequencing data files. This has the advantage of significantly decreased processing times, as well as not creating large intermediate files between processing steps, unless desired by the user. VDJPipe is typically the first step in an immunosequencing analysis workflow followed by assignment of V, D, and J gene segments to each sequence with subsequent repertoire annotation and characterization, and it has been successfully applied to analyzing human immune receptor sequences from B and T cells [8–10].

## Implementation

VDJPipe accepts as input a JSON-formatted configuration file that specifies the set of input sequencing data files and the sequence of processing tasks to be performed on those files. VDJPipe accepts both single-end and paired-end nucleotide sequence read files, and it requires quality scores that can be provided in either a FASTQ file or a FASTA/QUAL file pair. Input sequence files, compressed in gzip or bz2 format, can be read by VDJPipe without being decompressed. Paired-end reads can be initially merged into a single sequence based upon alignment of the overlapping nucleotides, but operations, such as quality filtering, can also be performed on each paired-end read file individually with reads being merged afterward. VDJPipe produces FASTA-formatted output files of processed reads for input into V(D)J assignment software, such as IgBlast.

### Sequence read filtering and statistics

VDJPipe provides many common operations, such as filtering based on nucleotide-level quality and average quality score, ambiguous base calls, homopolymer detection, and sequence length. Each operation has parameters to specify minimum, maximum, or window range values for applying the filter. VDJPipe can calculate various statistics at one or more steps along the processing pipeline. These statistics include nucleotide composition along the length of the read, GC-content histogram, sequence length histogram, average quality score histogram, and base call quality along the length of the read. Generating statistics at multiple points in the pipeline makes it easy to compare how the sequence read composition changes before and after each filtering operation.

### Barcode demultiplexing

It is a common practice with some next generation sequencing studies to multiplex multiple biological samples into a single sequencing run by attaching an identical set of short nucleotide barcodes to all sequences in a given sample. The barcode set is unique for each sample and can be used to demultiplex (split) the combined sequences produced in a single sequencing run back into individual sample files. VDJPipe supports any number of barcodes with its *match* operation, and barcode sequences with their sample identifier are specified in a separate FASTA file. Matching can be performed on the forward or reverse strand, can be limited to a specific search window, and can be a gapped or non-gapped alignment with either a minimal score or a maximum number of mismatches defining a successful match. The *match* operation uses the standard Smith-Waterman local alignment algorithm [11] with a substitution matrix that scores matches and mismatches (+2 for match and −2 for mismatch, or 0 for match and +1 for mismatch if only counting mismatches). The matching barcode can be trimmed from the sequence if desired, sequences without a matching barcode can be excluded, and each sequence is tagged with its barcode identifier allowing it to be used in later operations. VDJPipe can handle multiple combinatorial barcodes, such as are used in single-cell sequencing protocols [12], with multiple match operations or with the barcode combinations specified in a CSV file.

### 5′ and 3′ primer matching

Immunosequencing typically uses a targeted PCR protocol with a panel of 5′ (V region) and 3′ (J or C region) primers to capture the genes of interest. Other protocols use 5′ RACE, which eliminates the 5′ primer. As with barcodes, VDJPipe’s *match* operation can be used to recognize the primer sequences, trim them from the sequence if desired, and tag each sequence with the primer identifier for use in later operations. Primer sequences are specified in a separate FASTA file.

### Duplicate reads

Adaptive immune cells can undergo clonal expansion which generates daughter cells with identical V(D)J recombination sequences (though some B cells also undergo somatic hypermutation that can alter the sequence). When sequencing a large number of immune cells, these clones appear as duplicate sequences within the data. Duplicates also appear as a consequence of PCR amplification during sample preparation. Collapsing duplicate reads greatly shrinks the data size and can speed up downstream analyses. However, duplicate read checking in standard tools focused on genome sequencing or RNA-seq assumes only a portion of the sequence needs to be identical in order for the read to be marked as a duplicate [6], but this assumption is invalid for immune repertoire sequencing. Many V, D, and J gene segments are highly similar, and allelic variations present in individuals may only differ by a few nucleotides. Therefore, it is important that the complete read sequence be checked. The standard n-gram hash table approach cannot be used, however, because immune receptor read lengths are typically greater than 250 nucleotides. Thus, VDJPipe utilizes a suffix tree data structure to store the unique sequences found while processing the data. Furthermore, VDJPipe recognizes the sample barcode demultiplexing and collapses duplicate reads within each sample separately. A report of the duplicate count for each read is provided as part of the output.

## Results

We compare the performance of VDJPipe v0.1.7 with that of another software tool specialized for immunosequencing data, pRESTO v0.5.3 [13]. pRESTO has an alternative design of providing a set of Python scripts, each of which performs one step in the pre-processing workflow. For comparison, we use two example data sets provided by pRESTO [14, 15] and publically available from SRA under accession ID: ERP003950 and SRX190717. The first data set is Illumina MiSeq 2 × 250 stranded paired-end reads from RNA isolated from antibody-secreting mouse cells with primers for the amplification of full-length IgG heavy chain variable regions [14]. The second data set is Roche 454 reads from B-cell RNA isolated from PBMC for human patients across multiple time points before and after exposure to the influenza vaccine [15]. For the first data set, processing steps include merging the paired-end reads into a single read sequence, quality filtering, 5′ and 3′ primer matching, and collapsing duplicate reads. For the second data set, processing steps include length, homopolymer and quality filtering, generating compositional statistics, barcode demultiplexing, 5′ and 3′ primer matching, and collapsing duplicate reads. Together, these two data sets test all the main functions provided by VDJPipe (Table 1). We use the execution scripts provided by pRESTO for the examples but comment out the miscellaneous steps of parsing log files and compressing intermediate files at the end. The JSON input files used for VDJPipe are provided in the sample_data directory in the source code repository. Tests were run on a quad core Linux computer; VDJPipe only utilizes one processing core, while pRESTO is able to use all four cores.

Tables 2 and 3 show a comparison of execution times for both tools with different size input files for each data set, respectively. We find that VDJPipe is able to complete all processing steps in less than 10% the time required by pRESTO.

**Table 2:** Execution times for the Greiff et al., [14] data set

|Sequences|	VDJPipe (1 core)	pRESTO (4 cores)
25,000	5.7 s (0.1 s)	1 m 43.2 s (2.5 s)
250,000	1 m 7.5 s (0.5 s)	17 m 35.1 s (15.6 s)
1,085,869	8 m 22.5 s (4.6 s)	89 m 14.9 s (2 m 18.5 s)

Execution times are the average of ten runs with the standard deviation in parentheses

**Table 3:** Execution times for the Jiang et al., [15] data set

|Sequences|	VDJPipe (1 core)	pRESTO (4 cores)
50,000	6.5 s (0.2 s)	1 m 30.5 s (1.2 s)
277,826	40.3 s (0.2 s)	15 m 5.1 s (4.9 s)

Execution times are the average of ten runs with the standard deviation in parentheses

## Conclusion

VDJPipe is a flexible, high-performance tool for performing a variety of pre-processing operations on immune repertoire sequencing data. Written in C++ and utilizing a pipelined design, VDJPipe can efficiently perform all its operations with just a single pass over the input data file. This results in significantly decreased processing times. This has the additional benefit of not creating large intermediate files (unless desired by the user) between each processing step. Future enhancements include support for multiprocessing and building consensus sequences from unique molecular identifiers.

## Availability and requirements

**Project name:** VDJPipe

**Project home page:** https://vdjserver.org/vdjpipe/index.html

**Source code repository:** https://bitbucket.org/vdjserver/vdj_pipe

**Docker image:** https://hub.docker.com/r/vdjserver/vdj_pipe

**Operating system(s):** Platform independent

**Programming language:** C++

**License:** GNU GPL

Any restrictions on use by non-academics: no restrictions. The data sets analyzed in this study are publicly available and described in [14, 15]. The VDJPipe input configuration files used for these two data sets are available in the source code repository in the sample_data directory.
